# Detoxification of Ochratoxin A by *Bacillus amyloliquefaciens* MM28: Whole-Genome Sequencing and Safety Evaluation of a Novel Probiotic Strain

**DOI:** 10.3390/foods15060976

**Published:** 2026-03-10

**Authors:** Yanyan Jia, Jing Guo, Yixin Shen, Chengshui Liao, Songbiao Chen, Ke Ding, Zuhua Yu

**Affiliations:** 1 Laboratory of Functional Microbiology and Animal health, College of Animal Science and Technology, Henan University of Science and Technology, Luoyang 471023, China; 9903945@haust.edu.cn (Y.J.); 18317545493@163.com (J.G.);; 2 Luoyang Key Laboratory of Live Carrier Biomaterial and Animal Disease Prevention and Control, Luoyang 471023, China; 3 Ministry of Education Key Laboratory for Animal Pathogens and Biosafety, Zhengzhou 45000, China; 4 College of Animal Science and Technology, Henan Institute of Science and Technology, Xinxiang 453003, China

**Keywords:** biodegradation, genomic analysis, mycotoxin, probiotics, mice

## Abstract

Ochratoxin A (OTA), a secondary metabolite produced by *Penicillium* and *Aspergillus* species, contaminates food and feed globally, posing serious threats to both livestock and human health. Among current detoxification strategies, probiotic-based degradation of OTA has emerged as a key research focus. This study aimed to isolate safe probiotic strains with high OTA-detoxifying efficacy to support their potential application in feed and food industries. A total of 57 bacterial strains were isolated from environmental samples, including soil, moldy feed, and animal feces. Among these, a novel strain identified as *Bacillus amyloliquefaciens* MM28 demonstrated strong OTA-degrading activity, removing 86.31% of OTA (0.4 µg/mL) within 48 h. Whole-genome analysis indicated that *B. amyloliquefaciens* MM28 harbors functional genes related to glucose metabolism, membrane transport, and properties associated with antibacterial, antioxidant, and immunomodulatory activities, suggesting multiple beneficial traits. In a 28-day chronic exposure study, mice were administered *B. amyloliquefaciens* MM28 via gavage (1 × 10^8^ CFU/mL). Results showed that both female and male mice in the MM28 group exhibited higher body weight and improved growth performance compared to the PBS control group. Furthermore, intestinal morphology was enhanced in the MM28 group, as indicated by greater villus length and villus-length-to-crypt-depth ratio. The expression of proinflammatory cytokines was also reduced in the treated animals. Moreover, analysis of gut microbiota composition revealed that MM28 supplementation led to an increased abundance of *Bacteroides* and *Desulfovibrio*, alongside a reduction in *Lachnospira* and *Oscillospira*. In conclusion, this study demonstrates that *Bacillus amyloliquefaciens* MM28 is a safe and efficient strain capable of degrading OTA. These findings highlight its promising potential as a biological detoxifying agent in food and feed industries.

## 1. Introduction

Ochratoxins are secondary metabolite mycotoxins mainly produced by *Penicillium* and *Aspergillus* species [1]. The ochratoxin family consists of over twenty structurally related subtypes, of which OTA, OTB, and OTC are the most prominent [2]. Among these, OTA exhibits the highest toxicity and is of greatest concern. It frequently contaminates a variety of food commodities such as cereals, meat, dairy products, wine, coffee, infant formula, and bottled water, constituting a serious threat to human health [3,4,5]. After animals ingested OTA-contaminated feed, OTA accumulated in their tissues. Consumption of food derived from OTA-contaminated animal or plant sources may then trigger various adverse effects. Consumption of foods tainted with OTA is associated with multiple adverse health outcomes, including carcinogenic, hepatotoxic, nephrotoxic, and immunotoxic effects [6,7,8,9]. In recognition of its hazardous nature, OTA has been designated as a Group 2B probable human carcinogen by the International Agency for Research on Cancer (IARC) and the World Health Organization (WHO) [10]. Consequently, there is a pressing demand for the development of safe and efficient strategies to detoxify OTA in contaminated products.

Presently, there are three primary approaches for OTA detoxification: physical, chemical, and biological. Physical adsorption and chemical detoxification methods are often costly and tend to alter the nutritional profile of feed and food, thereby posing potential safety risks. Moreover, their application is often hindered by limitations such as suboptimal efficacy, high equipment costs, significant impacts on product quality, and low consumer acceptability [11,12]. Compared to conventional methods, biological detoxification presents a more efficient and environmentally friendly alternative, making it a highly promising approach [13,14]. Biological detoxification employs microbial and enzymatic approaches. Among these, the use of living organisms such as bacteria represents one viable option. The degradation of OTA by probiotics, for instance, can be attributed to three main mechanisms: antagonistic inhibition, bacterial adsorption, and bio-enzymatic degradation [15,16]. Antagonistic bacteria, such as those belonging to the genus *Bacillus*, can compete with toxigenic strains, partially inhibiting their growth. For example, short peptides secreted by *Bacillus subtilis* not only inhibit the growth of *Phytophthora capsici* and *Penicillium chrysogenum* but also reduce the activity of β-1,3-glucan synthase (GS) produced by these molds [17]. β-1,3-Glucan constitutes a critical structural component of the fungal cell wall. Inhibition of β-1,3-GS compromises cell wall integrity, resulting in localized lysis at hyphal tips or budding sites, subsequent leakage of intracellular contents, and ultimately fungal cell death [17].

Bacterial adsorption mainly relies on cell wall components—such as mannoproteins, peptidoglycan, and small-molecular-weight proteins—to remove toxins. For example, *Saccharomyces cerevisiae* ŁOCK 0119 (2 × 10^7^ CFU/mL) can adsorb OTA via its cell wall and surface charge, achieving a degradation rate of 52.7% within 24 h against an initial OTA concentration of 100 μg/mL [18]. Enzymatic degradation, on the other hand, involves the use of extracellular or intracellular bacterial enzymes that convert OTA into non-toxic, lower-molecular-weight metabolites. Zheng et al. [19] found that *Alcaligenes faecalis* ANSA176 isolated from donkey intestinal contents, degraded 97.43% of OTA within 12 h under conditions of pH 6.0–9.0 and 37 °C. This strain acts by hydrolytically cleaving the amide bond of OTA, yielding low-toxicity OTα. In addition, certain probiotic strains detoxify OTA through a combination of adsorption and degradation. For instance, *B. amyloliquefaciens* ASAG1 removes up to 99.7% of OTA by cell wall adsorption or degradation. A carboxypeptidase isolated from *B. amyloliquefaciens* has been shown to hydrolyze OTA into low-toxicity OTα and L-β-phenylalanine (Phe), effectively eliminating its toxicity [20]. Therefore, probiotics detoxifying OTA has become a current research hotspot and holds great potential.

This study aimed to isolate bacteria capable of effectively degrading OTA from soil, moldy feed, and animal feces collected on university grounds. The most promising strain was selected and subjected to genome sequencing and further genomic analyses to identify functional genes. Its OTA-degrading capacity was assessed, followed by in vivo safety evaluations to determine its potential as a probiotic.

## 2. Materials and Methods

### 2.1. Bacterial Strain, Culture Media, and Chemicals

All of isolated-strains were isolated from soil, moldy feed, and animal feces in Luoyang, China, and preserved in glycerol stocks at −80 °C in the Laboratory of Livestock and Poultry Health and Functional Microbiology, Henan University of Science and Technology. These strains were routinely cultured on Lysogeny Broth (LB) medium (10 g/L tryptone, 5 g/L yeast extract, 10 g/L NaCl, and 15 g/L agar; tryptone, yeast extract, and agar were purchased from Oxoid, and NaCl from Sinopharm Chemical Reagent Co., Ltd., Shanghai, China) at 37 °C. For OTA analysis, OTA standard substances were obtained from Qingdao Purenbang Biotechnology Engineering Co., Ltd., Qingdao, China, and detection was performed using an ELISA kit (for bacterial screening) and an OTA immunoaffinity column (for high-performance liquid chromatography analysis), both sourced from HUAAN MAGNECH Biotechnology Co., Ltd., Beijing, China.

### 2.2. Isolation and Screening of OTA-Degrading Strains

To isolate OTA-degrading microorganisms, environmental samples (e.g., soil, moldy feed, animal feces) were collected from university grounds. Each sample (1 g) was diluted 1:9 with sterile water and vigorously shaken. Aliquots were spread on solid LB medium (containing agar) and inoculated into liquid LB medium (without agar), with or without 0.4 µg/mL OTA. Solid plates were incubated statically at 37 °C in an electrically heated constant-temperature incubator (Shanghai Xinmiao Medical Instrument Manufacturing Co., Ltd., Shanghai, China), and liquid cultures were incubated at 37 °C with 180 rpm for 24 h. Single colonies were isolated from the solid medium using an inoculation loop and purified to obtain pure cultures, then subjected to secondary screening in OTA-supplemented LB liquid medium. The OTA degradation rate was determined by ELISA to select strains with high degradation efficiency. The residue of OTA was detected according to the instructions in the ELISA kit. The degradation rate was calculated using the following formula. OTA degradation rate: OTA degradation rate/% = [(OTA content in the control group-OTA content in the treatment group)/OTA content in the control group] × 100%.

### 2.3. Identification of OTA-Degrading Strain

The isolated strain was cultured on LB plates and purified, and its morphological characteristics were examined. Bacterial smears were prepared, air-dried, heat-fixed, and subjected to Gram staining: crystal violet (1 min), iodine (1 min), decolorization with 95% ethanol, and counterstaining with safranin (30–60 s). Gram-positive cells appeared purple, and Gram-negative cells appeared pink under light microscopy. For molecular identification, genomic DNA was extracted using a bacterial genome kit (TIANGEN, Beijing, China). The 16S rDNA gene was amplified with primers 27F and 1492R under the following conditions: 94 °C for 4 min; 35 cycles of 94 °C for 30 s, 55 °C for 30 s, and 72 °C for 1 min; and a final extension at 72 °C for 10 min. The obtained sequence was compared against the NCBI 16S rDNA database. A phylogenetic tree was constructed using the Maximum Likelihood method in MEGA 11.0.1318 with 14 closely related sequences and visualized using Chiplot (https://www.chiplot.online/tvbot.html, accessed on 13 September 2024).

### 2.4. Detection of OTA-Degradation Ability by HPLC

This experiment was performed in strict accordance with the Hua’an Maike OTA purification column protocol. HPLC (fluorescence detector, Waters, 2695), centrifuge, ultrasonic instrument, and incubator were calibrated. An OTA-specific column (Hua’an Maike, Beijing, China, HCM0750A) and a C18 column (5 µm, 4.6 × 150 mm) were activated and equilibrated. PBS, eluent, and OTA standard (Pribolab Biotech Co., Ltd., Qingdao, China) were prepared, and consumables were preconditioned. A 100 µg/mL OTA stock solution was accurately prepared and stored in the dark.

For degradation assays, MM28 was cultured in LB at 37 °C for 12 h. Cells were harvested (12,000× *g*), washed, resuspended in PBS, and lysed by ultrasonication. Then, 996 µL of lysate was mixed with 4 µL of OTA stock solution (final concentration 0.4 µg/mL) and incubated at 37 °C for 48 h. After incubation, samples were extracted with PBS, ultrasonicated, centrifuged, and subjected to immunoaffinity column cleanup (Hua’an Maike, Beijing, China). The eluate (200 µL) was filtered (0.22 µm) and injected (50 µL) into the HPLC system (OTA retention time: 12.481 min).

Chromatographic separation was performed on a C18 column at 35 °C with isocratic elution (acetonitrile/water/glacial acetic acid, 48:51:1, *v*/*v*/*v*) at 1.0 mL/min. Fluorescence detection was set at λex 330 nm and λem 460 nm. Quantification was based on a calibration curve (R^2^ ≥ 0.999).

### 2.5. Genome Sequencing and Assembly of B. amyloliquefaciens MM28

Genomic DNA of *Bacillus amyloliquefaciens* MM28 was extracted using the Tiangen DP302 kit following the manufacturer’s protocol. Briefly, lysed cells were treated with proteinase and RNase to remove proteins and RNA. DNA was precipitated with isopropanol, washed with ethanol, and dissolved in elution buffer, then stored at −20 °C. Whole-genome sequencing was performed by Sangon Biotech (Shanghai) on an Illumina NovaSeq 6000 platform with a paired-end library (2 × 150 bp) [21]. Raw reads were quality-filtered using FastQC 0.11.2 [22]; those containing ≥10% ambiguous bases (N), ≥40% bases with Phred score ≤ 15, or adapter sequences were removed, and only reads trimmed to ≥35 nt were retained. High-quality reads were assembled *de novo* using SPAdes 3.5.0 [23]. Gaps in the resulting contigs were closed with GapFiller 1.11 via local reassembly of paired-end reads [24]. The assembly was further refined using PrInSeS G 1.0.0 to correct base errors and indels by alignment with the original reads [25]. Gene prediction and annotation—including coding sequences, tRNAs, rRNAs, and other genomic features—were conducted using Prokka 1.10 [26], with *B. amyloliquefaciens* DSM7 (GenBank: NC_014551.1) as the reference.

### 2.6. Functional Annotation of the B. amyloliquefaciens MM28 Genome

The *B. amyloliquefaciens* MM28 genome was annotated with Prokka and visualized as a circular map using CGViewer [27]. Phylogenetic relatedness and functional attributes of homologous sequences were evaluated by aligning the genome against the NCBI Non-Redundant (NR) database. Protein sequences were functionally classified through BLAST+ searches against the COG (https://www.ncbi.nlm.nih.gov/COG/), SwissProt, and TrEMBL databases [28,29]. Gene Ontology (GO: http://www.geeontology.org) terms were assigned based on corresponding annotations from SwissProt (https://www.uniprot.org) and TrEMBL. Additionally, Kyoto Encyclopedia of Genes and Genomes (KEGG: http://www.kegg.jp) pathway annotations were acquired via the KEGG Automatic Annotation Server (KAAS) [30,31].

### 2.7. Prediction of Genes Encoding CAZymes and Secondary Metabolites in B. amyloliquefaciens MM28

To identify specific functional genes, the predicted secretory proteins of *B. amyloliq uefaciens* MM28 were aligned against the carbohydrate-active enzymes (CAZymes) database using dbCAN to annotate CAZymes [32,33]. Additionally, secondary metabolite biosynthetic gene clusters were predicted using the antiSMASH online tool [34].

### 2.8. Probiotics Genetic Analysis

The genomic sequences of the probiotic strains were obtained from the NCBI database. Using TBtools 1.115, the complete genome of *B. amyloliquefaciens* MM28 was aligned against the retrieved reference sequences.

### 2.9. Animal Experimental Design

A total of twelve 20-day-old Kunming mice were randomly assigned to two experimental groups: a PBS control group and a *B. amyloliquefaciens* MM28-treated group, each comprising three males and three females. Throughout the 28-day experiment, the PBS group was administered 0.2 mL of PBS daily via oral gavage, whereas the treated group received an equivalent volume of bacterial suspension (1 × 10^8^ CFU/mL) following the same procedure. General health status was assessed daily, and body weight was monitored over the entire study duration. On the 28th day of the experiment, after the eyeball was sacrificed for blood collection, the organ indices were recorded.

### 2.10. Detection of Serum Antioxidant Indicators

At the end of the 28-day feeding period, approximately 1 mL of blood was obtained from each animal without anticoagulant treatment, allowed to clot for 2 h, and centrifuged at 3000× *g* for 10 min at 4 °C. Serum samples were processed strictly following the manufacturer’s instructions for malondial-dehyde (MDA), glutathione peroxidase (GSH-Px), and total superoxide dismutase (T-SOD) assay kits (Jiancheng, Nanjing, China), which involved sample pretreatment, reagent incubation, and colorimetric reaction. Subsequently, the absorbance of each reaction mixture was measured using a microplate reader. The content and activity of each target index were quantified using the standard formulas supplied with the kits, so as to conduct the assessment of serum antioxidant status.

### 2.11. Quantitative Real-Time PCR

After routine orbital blood collection and euthanasia, tissue samples from each group were preserved in RNA fixative at −20 °C. Total RNA was extracted from jejunal tissue using Trizol (Beyotime). Briefly, tissue fragments were homogenized with Buffer RL and 3–5 grinding beads (2 min), then passed through a gDNA Filter Column (12,000 rpm, 2 min). The filtrate was mixed with an equal volume of 70% ethanol, transferred to an RA spin column, and centrifuged (12,000 rpm, 1 min). The column was sequentially washed with Buffer RW1 (500 µL) and Buffer Rw2 (600 µL, twice), air-dried, and eluted with 30~50 µL RNase-Free H_2_O. RNA concentration was measured using a nucleic acid analyzer (Thermo Fisher Scientific, Waltham, MA, USA). cDNA was synthesized on ice using a One-Step RT system (42 °C for 15–30 min, 85 °C for 5 min).

qRT-PCR was performed on a 7500 Real-Time PCR System (Applied Biosystems, Foster City, CA, USA) using Premix Ex Taq (TaKaRa) to measure the gene expression of selected pro-inflammatory cytokines. Each 20 µL reaction contained 10 µL 2× Premix, 1 µL each of forward and reverse primers, 1 µL cDNA, and 8 µL dH_2_O. Cycling conditions: 95 °C for 30 s, followed by 40 cycles of 95 °C for 5 s and 60 °C for 34 s. All reactions were run in triplicate and repeated three times.

### 2.12. Histopathology

After the routine blood collection from the eyeball, samples of 1–1.5 cm from each intestine were fixed in 4% para-formaldehyde. The HE staining and slide photography were completed at Chengdu LiLai Biotechnology Co., Ltd., Chengdu, China.

### 2.13. Analysis of Gut Microbial Diversity

Cecal contents were collected from five randomly selected mice per group for high-throughput sequencing. The dry ice–preserved samples were stored under controlled conditions and shipped to Shanghai Ouyi Biomedical Technology Co., Ltd., Shanghai, China. for DNA extraction and subsequent quality control assessment. High-quality sequencing reads were generated and processed for operational taxonomic unit (OTU) clustering and taxonomic classification. Microbial alpha and beta diversity analyses—based on the resulting OTU profiles—were performed to evaluate both within-sample (alpha) and between-sample (beta) microbial community diversity.

### 2.14. Statistical Analysis

Statistical analysis was performed with SPSS 21.0 (IBM, Armonk, NY, USA). After confirming normality, continuous variables (presented as mean ± SEM) were compared using one-way ANOVA or *t*-tests, followed by Duncan’s post hoc test for multiple comparisons. Graphs were generated in GraphPad Prism 8.3.0 and Chiplot. Differences were considered statistically significant at * *p* < 0.05 (moderate) and ** *p* < 0.01 (high).

## 3. Results

### 3.1. Screening of OTA-Degrading Strains

A total of 57 bacterial strains were isolated from diverse environmental samples, including soil, moldy feed, and animal feces. Primary screening on a medium with coumarin as the sole carbon source identified seven strains capable of stable growth. These included two strains (MM43, MM15) from moldy feed, one (MM12) from animal feces, and four (MM9, MM25, MM28, MM39) from soil. The degradation efficiency of the strain towards OTA was detected using an ELISA kit (Table 1). Among them, strain MM28, which demonstrated the highest degradation rate, was selected for further study.

### 3.2. Identification of OTA-Degrading Strain

**Figure 1:**
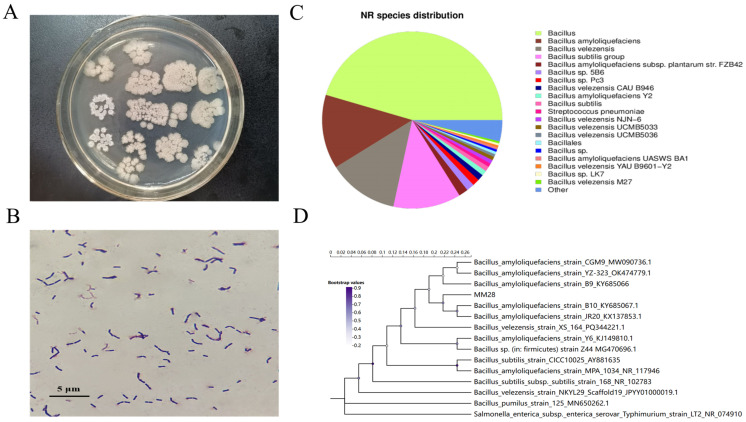
Morphological and molecular identification of *B. amyloliquefaciens* MM28. (**A**) Colony morphology; (**B**) Appearance under a microscopy after Gram stain (100×); (**C**) Pie chart of thehomological distribution of *B. amyloliquefaciens* MM28; (**D**) Phylogenetic tree of related strains based on the 16S rDNA gene sequence.

Further analyses revealed that MM28 is a Gram-positive bacterium, and produced a white film on the surface of liquid media in LB broth. On solid media, the colonies were large, white, and irregularly shaped, with smooth depressions in the center and irregular elevations around the periphery (Figure 1A,B).

The 16S rDNA sequence of MM28 comprises 1452 base pairs and has been deposited in GenBank under accession number ON202807.1. A BLAST analysis of the complete 16S rDNA sequence of strain MM28 identified *B. amyloliquefaciens* as its closest relative (Figure 1D). Consistent results were obtained through analysis of the NR database (Figure 1C). Therefore, based on molecular biological analysis, strain MM28 was identified as *Bacillus amyloliquefaciens*.

### 3.3. Mechanism of OTA Degradation by B. amyloliquefaciens MM28

**Figure 2:**
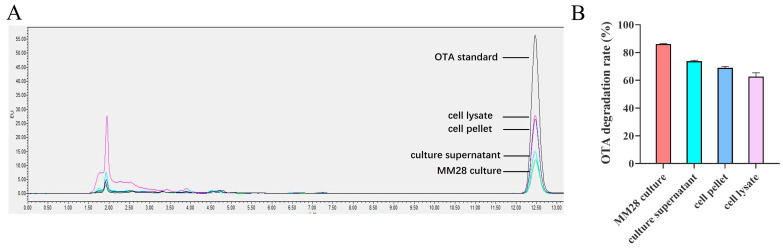
OTA residues were detected by HPLC. (**A**) The OTA degradation activity of each component of *B. amyloliquefaciens* MM28 by HPLC. (**B**) OTA degradation rate of each component. The assay was performed in triplicate and repeated three times.

The degradation of OTA by various fractions of *B. amyloliquefaciens* MM28 was quantified by HPLC. Notably, the whole bacterial culture and its culture supernatant achieved degradation rates of 86.31% and 73.02%, respectively. The cell pellet and cell lysates also showed significant degradation, with rates of 67.75% and 59.95% (Figure 2). These results reveal that OTA is detoxified by *B. amyloliquefaciens* MM28 via a combined pathway of cellular adsorption and enzymatic degradation.

### 3.4. Genomic Overview of B. amyloliquefaciens MM28

**Figure 3:**
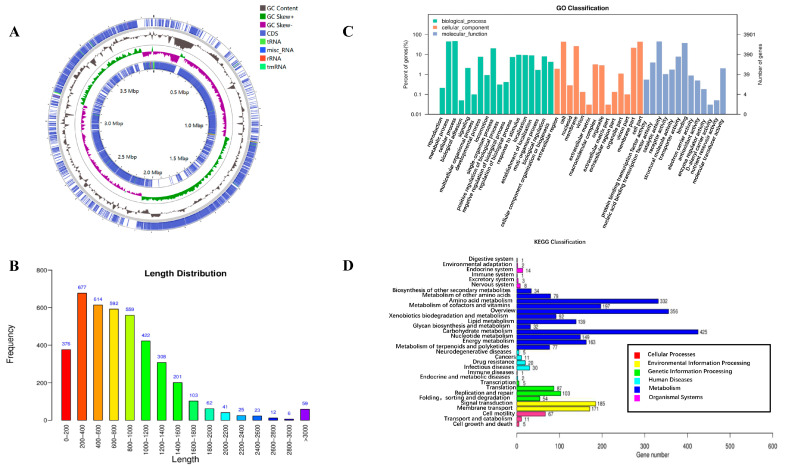
Whole-genome analysis of *B. amyloliquefaciens* MM28. (**A**) Genome circular map of *B. amyloliquefaciens* MM28. Circular representation of the *B. amyloliquefaciens* MM28 genome. Tracks (from outermost inward): 1 & 4 (blue), forward and reverse strand genes (CDS, tRNA, rRNA); 2: (black), GC content; 3: (purple/green), GC skew. (**B**) Length distribution of protein-coding genes. The histogram illustrates gene count (y-axis) per protein sequence length interval (x-axis). (**C**) GO annotation results of *B. amyloliquefaciens* MM28. (**D**) Taxonomic map for KEGG annotation of *B. amyloliquefaciens* MM28 genes.

The genomic features of *B. amyloliquefaciens* MM28 are summarized in Table 2. The genome contained coding genes totaling 3,974,535 bp, with individual lengths spanning from 56 to 16,302 bp (average: 817.69 bp). Most genes (200~1000 bp) accounted for the predominant size range (Figure 3B), and the average G+C content was 46.25%.

### 3.5. COG Functional Annotation of B. amyloliquefaciens MM28

All protein-coding genes were annotated against the Clusters of Orthologous Groups (COG) database. The resulting functional profile revealed that *B. amyloliquefaciens* MM28 possesses a substantial number of genes involved in amino acid transport and metabolism (E) and transcription (K), followed by carbohydrate transport and metabolism (G) (Table 3). An overview of the genome is presented in the annotated circular map (Figure 3A).

### 3.6. GO Functional Annotation of B. amyloliquefaciens MM28

To elucidate their functional roles, the protein-coding sequences of *B. amy loliquefaciens* MM28 were annotated against the Gene Ontology (GO) database. Based on this annotation, the encoded proteins were classified into the three standard GO categories: Cellular Component (CC), Biological Process (BP), and Molecular Function (MF).

GO annotation revealed that the genes of *B. amyloliquefaciens* MM28 are predominantly associated with metabolic and cellular processes, cellular components, binding, and catalytic activity, followed by antioxidant and enzyme regulator activities (Figure 3C). This functional profile suggests that the strain possesses a robust metabolic capacity, secretes a diverse array of enzymes, and exhibits significant antioxidant activity.

### 3.7. KEGG Functional Annotation of B. amyloliquefaciens MM28

KEGG database mapping of the *B. amyloliquefaciens* MM28 genome identified 2861 genes. The annotation revealed a significant enrichment of genes in pathways for carbohydrate metabolism, membrane transport, and amino acid metabolism (Figure 3D). These genetic features suggest that the strain possesses a robust foundational metabolism, supportive of efficient membrane transport and rapid growth potential.

### 3.8. CAZy-Based Functional Annotation of B. amyloliquefaciens MM28

**Figure 4:**
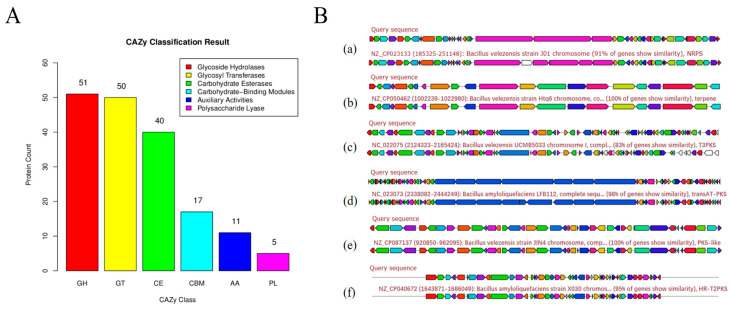
Carbohydrate-active enzymes and secondary metabolites of *B. amylolique faciens* MM28. (**A**) Distribution of the carbohydrate active enzymes (CAZy) in the genome of B. amyloliquefaciens MM28. (**B**) Analysis of secondary metabolites of *B. amyloliquefaciens* MM28: (a) NRPS; (b) Terpene; (c) T3PKS; (d) trabsAT-PKS; (e) PKS-like; (f) hR-T2PKS.

CAZymes, key players in carbohydrate metabolism, are classified in the CAZy database into five major classes: glycoside hydrolases (GH), glycosyltransferases (GT), carbohydrate esterases (CE), polysaccharide lyases (PL), and auxiliary activities (AA). Genomic analysis of *B. amyloliquefaciens* MM28 identified 174 CAZymes, distributed as 51 GH, 50 GT, 40 CE, 17 CBM, 11 AA, and 5 PL (Figure 4A). Glycoside hydrolases and glycosyltransferases were the predominant types, accounting for the two most abundant categories at 29.3% and 28.7% of the total, respectively.

### 3.9. Identification of Secondary Metabolite Biosynthetic Gene Clusters in B. amylolique faciens MM28

In silico analysis of *B. amyloliquefaciens* MM28 genome revealed a diverse arsenal of biosynthetic gene clusters (BGCs) with putative antibacterial functions. The strain harbors gene clusters encoding non-ribosomal peptide synthetases (NRPS), terpenoids (exhibiting 100% similarity to known terpenoid BGCs), and four distinct polyketide synthase (PKS) clusters. Notably, one of these PKS clusters demonstrated 100% identity to a known polyketide biosynthesis gene cluster (Figure 4B).

### 3.10. Analysis of Probiotic-Associated Genomic Features in MM28

To identify probiotic traits in the *B. amyloliquefaciens* MM28 genome, we first retrieved gene sequences associated with probiotic functions from the NCBI database. The whole-genome sequence was then screened against this custom dataset using local BLAST alignment in TBtools 1.115. This analysis revealed nine genes encoding putative probiotic properties (Table 4).

### 3.11. Safety Assessment of B. amyloliquefaciens MM28 In Vivo

#### 3.11.1. Effects of *B. amyloliquefaciens* MM28 on Body Weight, Feed Intake, and Organ Indices in Mice

**Figure 5:**
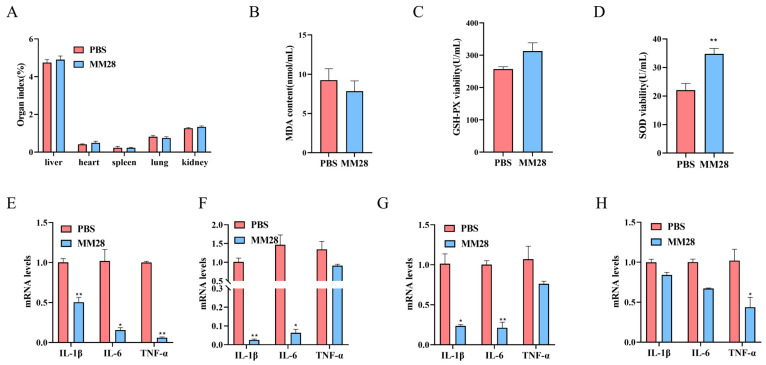
Safety assessment of *B. amyloliquefaciens* MM28 in mice. (**A**) Indices of each organ of experimental mice. Serum levels of oxidative stress markers in mice. (**B**) MDA; (**C**) GSH-PX; (**D**) SOD. Expression of key inflammatory factors in mice: (**E**) spleen; (**F**) duodenum; (**G**) the jejunum; (**H**) the ileum. Data are presented as mean ± SEM. * *p* < 0.05, ** *p* < 0.01 vs. control group.

Throughout the study, all mice remained healthy with smooth coats and normal activity; no mortality occurred. Gross examination at necropsy revealed no evidence of organ damage or hemorrhagic lesions. Mice administered *B. amyloliquefaciens* MM28 exhibited a significantly higher weight gain rate compared to the PBS group (*p* < 0.05; Table 5). Moreover, although male mice showed a greater weight gain than females, no significant differences in organ indices were observed between the treatment and control groups (*p* > 0.05; Figure 5A). These results collectively indicate that *B. amyloliquefaciens* MM28 promotes growth in mice without adversely affecting organ development.

#### 3.11.2. Effects of *B. amyloliquefaciens* MM28 on Antioxidant Levels in Mice

To evaluate the effect of *B. amyloliquefaciens* MM28 on oxidative stress, we measured serum levels of malondialdehyde (MDA), superoxide dismutase (SOD), and glutathione peroxidase (GSH-PX) in treated mice. Compared with the control group, the MM28 group showed a trend toward reduced serum MDA content (*p* > 0.05) and increased activities of SOD and GSH-PX. Notably, the increase in SOD activity was significant relative to the PBS group (*p* < 0.05), whereas the increase in GSH-PX activity did not reach statistical significance (Figure 5B–D).

#### 3.11.3. Effect of *B. amyloliquefaciens* MM28 on Inflammatory Cytokines in Mice

The anti-inflammatory effect of *B. amyloliquefaciens* MM28 was further evaluated by measuring key pro-inflammatory cytokines (IL-1β, TNF-α, IL-6). In the spleen, all three cytokines were significantly suppressed in the MM28 group compared to the control (*p* < 0.05). In the duodenum and jejunum, IL-1β and IL-6 levels were significantly lower (*p* < 0.05), whereas TNF-α expression remained unchanged (*p* > 0.05). Conversely, in the ileum, only TNF-α was significantly reduced (*p* < 0.05), with no significant differences in IL-1β and IL-6 (Figure 5E–H).

#### 3.11.4. Effect of *B. amyloliquefaciens* MM28 on Mouse Intestinal Tissue Morphology

**Figure 6:**
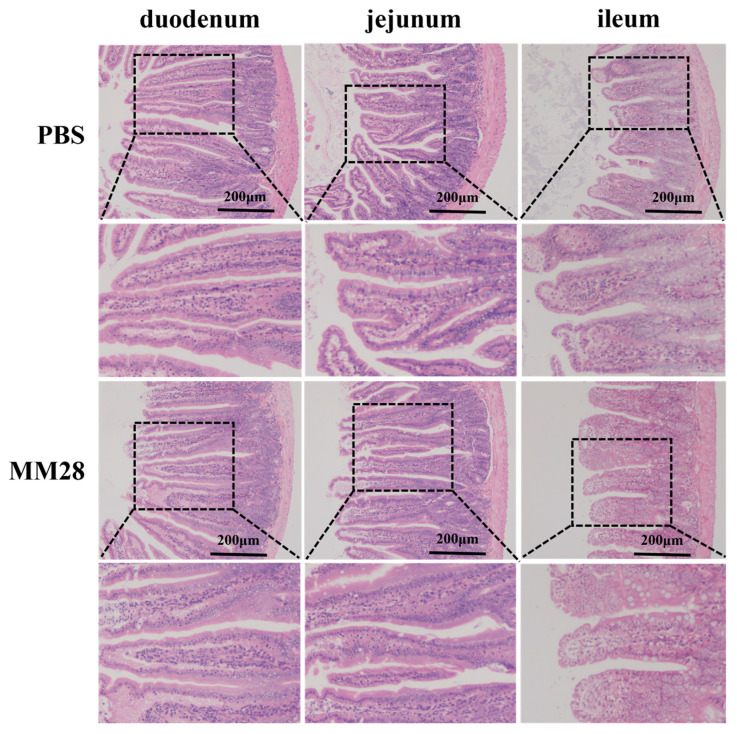
Representative morphology of mouse intestinal tissue following treatment with *B. amyloliquefaciens* MM28. Scale bar represents 200 μm (magnification: 100×).

The overall intestinal tissue morphology was uniformly normal in both groups, characterized by neatly arranged villi, intact epithelium, and smooth crypts without pathological proliferation (Figure 6).

Morphometric analysis indicated no significant differences in duodenal villus length, crypt depth, or villus-length-to-crypt-depth ratio (VCR) between *B. amyloliquefaciens* MM28 group and the control group. In the jejunum and Ileum, villus length was significantly increased in the MM28 group compared with the PBS control (*p* < 0.05). Furthermore, the VCR in various intestinal segments of the MM35 group was significantly higher than that in the PBS group (*p* < 0.05) (Table 6).

#### 3.11.5. Effect of *B. amyloliquefaciens* MM28 on the Microbial Diversity of the Mouse Cecum

**Figure 7:**
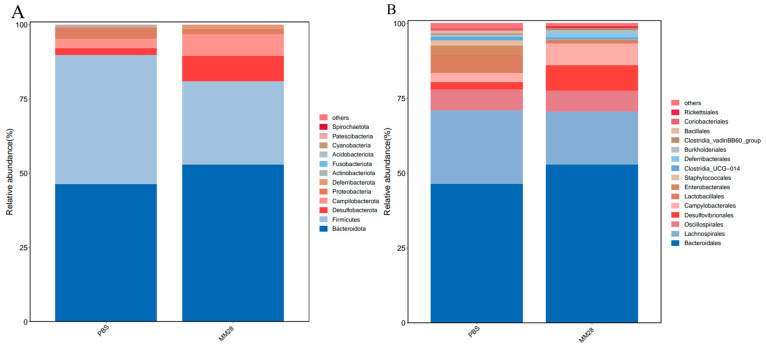
Gut microbial communities in mice gavaged with *B. amyloliquefaciens* MM28 and the prevalent taxa within these groups. Composition of the major bacterial phyla (**A**) and genera (**B**) in the sample.

To assess the impact of *B. amyloliquefaciens* MM28 on the cecal microbial composition, we analyzed the community structure at the phylum and genus levels. The predominant phyla across all samples were Firmicutes, Bacteroidetes, Desulfobacterota, and Campilobacterota (Figure 7A). No significant differences were observed in the abundance of these dominant phyla between the MM28 and PBS control groups (*p* > 0.05). At the genus level (Figure 7B), the six most abundant genera were *Bacteroides*, *Oscillospira*, *Lachnospira*, *Lactobacillus*, *Desulfovibrio*, and *Campylobacter*. Although not statistically significant (*p* > 0.05), the MM28 group exhibited trends of increased abundance in *Bacteroides* and *Desulfovibrio*, and decreased abundance in *Lachnospira* and *Oscillospira* compared to the control.

**Figure 8:**
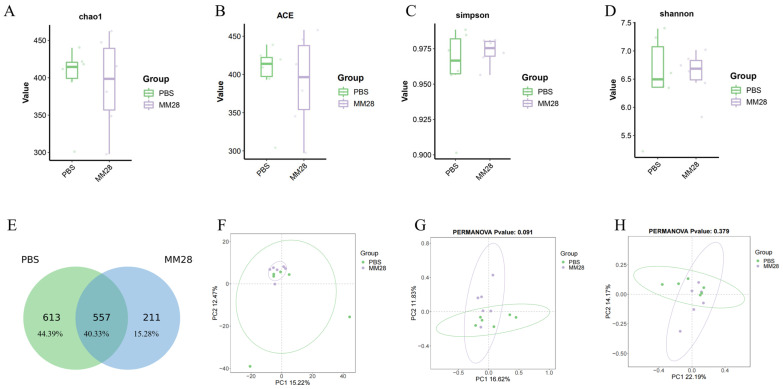
Diversity analysis of Alpha-microbial diversity and Beta-gut microbiome of mice gavaged with *B. amyloliquefaciens* MM28. (**A**) Chao1; (**B**) ACE; (**C**) Simpson; (**D**) Shannon; (**E**) Venn plots derived from OTU levels; (**F**) Plot of PCA analysis; (**G**) Plot of PCoA analysis (based on the binary Jaccard algorithm); (**H**) Plot of PCoA analysis (based on the unweighted unifrac algorithm).

A comprehensive assessment of cecal microbial alpha diversity was conducted using the Chao1 and ACE indices (richness) and the Shannon and Simpson indices (diversity). The results uniformly indicated no significant differences between the control and *B. amyloliquefaciens* MM28-treated groups (*p* > 0.05; Figure 8A–D). The Venn diagram revealed that shared OTUs between the control and experimental groups constituted 40.33% of the total (Figure 8E). Control-group-specific OTUs accounted for 44.39%, whereas those unique to the experimental group represented 15.28%. Principal component analysis further demonstrated that although the overall colony composition was highly similar between the PBS and *B. amyloliquefaciens* MM28 groups, the PBS group exhibited greater intra-group variation, whereas samples in the MM28 group were more clustered (Figure 8F). Consistent with this, both unweighted Jaccard and unweighted UniFrac analyses indicated no significant differences in species presence/absence or community structure between the two groups (Figure 8G,H).

## 4. Discussion

OTA remains a globally prevalent mycotoxin contaminating cereals, feeds, and agricultural products, with recent surveillance data revealing persistent contamination exceeding national regulatory limits in China and worldwide [35]. According to China’s national food safety standard GB 2761-2017 and feed hygiene standard GB 13078.2-2017, the maximum residue limits (MRLs) for OTA are set at 5 μg/kg in cereals and 100 μg/kg in feed materials respectively [36,37]. However, a 2025 investigation on feed ingredients and compound feeds across China reported that 1.1% of corn samples exceeded the feed MRL, with a maximum OTA concentration reaching 225.9 μg/kg (more than twice the limit), while corn gluten meal and poultry compound feeds showed median OTA levels exceeding 10 μg/kg [38]. The persistent OTA pollution exceeding regulatory thresholds poses severe threats to animal and human health, as this mycotoxin exhibits potent nephrotoxicity, carcinogenicity (Group 2B by IARC), immunotoxicity, and teratogenicity [35]. Therefore, there is an urgent need to develop innovative strategies for OTA mitigation that are not only highly specific and efficient, but also environmentally safe and minimally detrimental to product quality [39]. In this context, microbial detoxification has emerged as an effective, safe, and sustainable biological approach, representing both a major advancement and a guiding framework for OTA degradation [40].

*B. amyloliquefaciens* is approved by the Ministry of Agriculture and Rural Affairs of China as a feed microecological preparation [38]. It exhibits strong stress resistance, ease of cultivation, rapid growth, and high tolerance to the gastrointestinal environment of animals. This bacterium secretes a variety of extracellular enzymes that help regulate the gut microbiota, improve the digestion and absorption of feed, enhance immune function, and protect the intestinal epithelial barrier, thereby promoting gut health [41,42]. Furthermore, thanks to its ability to efficiently degrade mycotoxins and produce antimicrobial metabolites that inhibit pathogenic bacteria, it is also widely employed in the field of mycotoxin degradation [43,44]. In the present study, a novel Gram-positive bacterial strain, *B. amyloliquefaciens* MM28, with highly efficient OTA degradation capacity, was isolated from soil and initially identified through 16S rDNA sequencing. *B. amyloliquefaciens* MM28 degrades OTA through a synergistic interplay between bioadsorption and biodegradation, highlighting its potential as a promising feed detoxifier. In the future, we will further investigate the OTA degradation capability of this strain in vivo and elucidate the key degrading enzymes involved.

The functional genes in the *B. amyloliquefaciens* MM28 strain were comprehensively analyzed through whole-genome sequencing and bioinformatics analyses. The results showed that the genome size was 3,974,535 base pairs (bp), comprising 4080 coding genes, which account for 89.61% of the whole genome. The genomic data were compared and analyzed against various databases, including GO, COG, KEGG, and the NCBI NR database, to annotate the functional elements of the *B. amyloliquefaciens* MM28 strain genome. According to preliminary gene annotation, a significant number of genes in *B. amyloliquefaciens* MM28 are implicated in carbohydrate metabolism and membrane transport processes [45]. We predicted the carbohydrate genes of *Bacillus amyloliquefaciens* MM28 and identified 175 genes. Among them, the majority encodes glycosyl hydrolases (GH) and glycosyl transferases (GT) at 51 and 50, respectively. GT genes play a critical role in synthesizing surface structures that serve as targets for immune recognition [46]. The rich repertoire of GH and GT genes in *B. amyloliquefaciens* MM28 indicates its probiotic potential, which contributes to pathogen inhibition and immune modulation, ultimately enhancing mammalian host immunity. A range of secondary metabolites produced by *B. amyloliquefaciens* MM28 exhibit antifungal properties [47], broad-spectrum antibacterial activity, antiviral effects, antioxidant capabilities, and other activities such as immune regulation, anti-tumor effects, anti-parasitic properties, stimulation of intestinal peristalsis, and beneficial digestive effects [48,49,50]. Collectively, these results stimulate further in-depth exploration of the biological properties of *B. amyloliquefaciens* MM28. The findings of the present study revealed that *B. amyloliquefaciens* MM28 produces metabolites with antimicrobial, anti-inflammatory, and antioxidant properties, aiding the body in combating infections caused by pathogens. The present study identified nine genes popular in probiotics: dnaK, folC, msrB, fbp, dltA, dltB, dltD, rfbB, and clpC, which are primarily involved in antibacterial, immune response, and promoting the growth of beneficial microorganisms.

For a potential clinical OTA-degrading strain in the future, its safety is the primary consideration. Through genomic characterization, we aim to gain insight into its probiotic properties and safety, with further validation to be conducted via mouse experiments. These findings shows that oral administration of a specified dose of MM28 significantly enhances weight gain and reduces the feed conversion ratio in mice, indicating its role not only in stimulating growth but also in potentially improving feed utilization efficiency, consistent with findings by Bogsan et al. [51]. This suggests that *B. amyloliquefaciens* MM28 increases appetite in mice, enhancing the growth performance. The research conducted by Shifa A Bahaddad revealed that *Bacillus* species, as direct-fed microbials, exert significant anti-inflammatory effects by downregulating the expression of these pro-inflammatory cytokines, which is a crucial mechanism underlying their ability to improve gut health and replace antibiotic growth promoters in monogastric production [42]. *B. amyloliquefaciens* MM28 significantly reduced the expression levels of pro-inflammatory cytokines in the spleen and intestines of mice to varying degrees. Accordingly, it is suitable for supplementation in feeds.

Antioxidants are capable of effectively scavenging free radicals and aiding in the repair of cellular oxidative damage [52]. MDA is widely used as an indirect biomarker to evaluate systemic oxidative stress levels. Whereas SOD activity reflects antioxidant defense through its role in scavenging oxygen free radicals and preserving redox balance [53]. The results demonstrated that MM28 significantly increased SOD levels, while MDA content was downregulated to varying degrees compared with the control group. These findings suggest that *Bacillus amyloliquefaciens* MM28 enhances the antioxidant capacity of mice, thereby mitigating oxidative stress-induced damage. Furthermore, supplementation with *Bacillus amyloliquefaciens* MM28 led to varying degrees of downregulation in the mRNA expression of pro-inflammatory factors in both the spleen and intestines of mice. This indicates that MM28 may help maintain physiological homeostasis by suppressing the expression of inflammatory cytokines.

The supplementation of probiotics in feed can alter the structure of the animal’s gut microbiota [54]. Once colonized in the intestinal tract, probiotics exert colonization resistance against harmful bacteria through competitive inhibition [55]. Long-term feeding of MM28 did not induce significant pathological changes in the intestines of mice. Furthermore, in the jejunum and Ileum, villus length was significantly increased in the MM28 group compared with the PBS control. After feeding with *B. amyloliquefaciens* TL106 for 21 days, the height of the villi in the duodenum and jejunum, as well as the ratio of villus height to crypt depth, were higher than those in the control group [56]. The ratio of villus height to crypt depth in various intestinal segments of the MM28 group was significantly higher than that in the PBS group. Increasing gut microbiota richness and α-diversity by probiotic administration is essential for sustaining intestinal health. Higher microbial diversity strengthens colonization resistance against pathogens, boosts the abundance of beneficial bacteria, maintains intestinal microecological stability, and enhances the intestinal mucosal barrier. Meanwhile, an enriched gut microbiota optimizes nutrient digestion and absorption, modulates immune homeostasis, and alleviates excessive intestinal inflammation, thus improving growth performance and disease resistance in animals [40]. Gut microbiota analysis revealed that, compared to the control group, the MM28 group exhibited increased abundance of *Bacteroides* and *Desulfovibrio*, alongside decreased abundance of *Lachnospira* and *Oscillospira*. Previous studies have reported that *Bacteroides* strains, as potential probiotics, may exert anti-inflammatory and neuromodulatory effects [57]. These findings are closely aligned with the previously demonstrated probiotic effects of MM28. Principal component analysis revealed substantial intra-group variation in the PBS group, which may be attributable to individual differences among mice or environmental factors.

In summary, *Bacillus amyloliquefaciens* MM28, isolated from soil, was found to effectively degrade OTA. In animal studies, MM28 enhanced appetite and improved growth performance in mice. Furthermore, it contributed to physiological homeostasis by suppressing inflammatory cytokine expression and strengthening antioxidant defense. Analysis of gut microbiota composition revealed that MM28 supplementation led to an increased abundance of *Bacteroides* and *Desulfovibrio*, alongside a reduction in *Lachnospira* and *Oscillospira*. Although this study evaluated the degradation rate of OTA by probiotics, it did not include an in-depth analysis of the degradation products. Future research should strengthen this aspect to facilitate the clinical application of probiotics. Collectively, these findings highlight the potential of *B. amyloliquefaciens* MM28 as a promising functional feed additive for mitigating mycotoxin-related risks and improving animal health.

## Figures and Tables

**Table 1:** Degradation rate of OTA by various strains.

Strains	OTA Degradation Rate (%)	Strain Number	OTA Degradation Rate (%)
MM 9	16.61 ± 1.28	MM 25	78.37 ± 1.38
MM 28	86.31 ± 0.83	MM 12	31.82 ± 0.65
MM 15	60.12 ± 0.58	MM 43	25.32 ± 0.91
MM 39	45.60 ± 1.07		

**Table 2:** General genome features of *B. amyloliquefaciens* MM28.

Class	Number
Size (base)	3,974,535
G+C content (%)	46.25
Protein Coding Genes	4080
Min length (base)	56
Max length (base)	16,302
Average length (base)	872.91
Total coding gene (base)	3,561,475
Coding ratio (%)	89.61
tRNA	81
rRNA	10
Repeat Region	
Repeat Region Count	0
Total Repeat Region(base)	0
Repeat Ratio (%)	0

**Table 3:** COG categories of *B. amyloliquefaciens* MM28.

Code	Gene Num	Gene Ratio	Description
C	162	5.69	Energy production and conversion
D	36	1.26	Cell cycle control, cell division, chromosome partitioning
E	239	8.39	Amino acid transport and metabolism
F	85	2.98	Nucleotide transport and metabolism
G	219	7.69	Carbohydrate transport and metabolism
H	123	4.32	Coenzyme transport and metabolism
I	79	2.77	Lipid transport and metabolism
J	160	5.62	Translation, ribosomal structure and biogenesis
K	243	8.53	Transcription
L	123	4.32	Replication, recombination and repair
M	167	5.86	Cell wall/membrane/envelope biogenesis
N	22	0.77	Cell motility
O	86	3.02	Posttranslational modification, protein turnover, chaperones
P	159	5.58	Inorganic ion transport and metabolism

**Table 4:** Identification and characterization of probioticity genes in MM28.

Genes	Locus Tag	Functions	Identity (%)
*dnaK*	J5X95_RS01995	Stress Response and Immune System Interaction	99.18433931
*folC*	J5X95_RS02720	Support of Beneficial Microbiota and Cellular Growth and Division	96.9837587
*msrB*	J5X95_RS19555	Protein Repair and Maintenance	97.70114943
*fbp*	NG74_RS18755	Maintaining blood sugar levels and Energy Balance	97.6635514
*dltA*	J5X95_RS08060	Impacting the resistance of bacteria to certain antibiotics and antimicrobial peptides	98.21428571
*dltB*	J5X95_RS08065	Modulating immune responses and affect the bacterial resistance to certain cationic antimicrobial peptides and antibiotics	99.40778342
*dltD*	J5X95_RS08075	Modulating the immune response and Pathogen Recognition	98.89737065
*rfbB*	J5X95_RS07730	Polysaccharide Biosynthesis	98.31223629
*clpC*	J5X95_RS09815	Stress Response and Regulation of Cellular Processes	97.98602548

**Table 5:** Physiological growth indices in experimental mice.

	Index	Group	
		PBS	MM28
female mouse	Average initial weight (AW)	29.87 ± 0.80	31.13 ± 1.63
	Average weight (AW)	30.71 ± 0.54	33.35 ± 1.87
	weight gain percentage (%)	2.74 ± 1.24	6.65 ± 1.08 *
	Average daily weight gain (ADG)	0.03 ± 0.01	0.08 ± 0.02 *
	Mean daily feed intake (ADFI)	3.85	6.52
male mouse	Average initial weight (AW)	29.29 ± 0.91	30.83 ± 0.98
	Average weight (AW)	31.93 ± 0.63	40.10 ± 1.28
	weight gain percentage (%)	8.25 ± 1.96	23.13 ± 0.06 **
	Average daily weight gain (ADG)	0.09 ± 0.02	0.33 ± 0.01 **
	Mean daily feed intake (ADFI)	4.98	5.58

Note: data are presented as mean ± SEM deviation (n = 3), * 0.01 < *p* < 0.05, ** *p* < 0.01. PBS represents the control group.

**Table 6:** Morphometric analysis of the intestine in mice following *B. amyloliquefaciens* MM28 exposure.

		PBS	MM28
Duodenum	Villusheight, µm	494.295 ± 19.705	546.355 ± 3.364
	Crypt depth, µm	142.809 ± 4.221	147.001 ± 6.609
	VCR	3.464 ± 0.137	5.283 ± 1.059 ***
Jejunum	Villusheight, µm	347.909 ± 7.281	396.239 ± 11.652 *
	Crypt depth, µm	131.472 ± 12.736	116.124 ± 8.603
	VCR	2.710 ± 0.329	4.033 ± 0.660 *
Ileum	Villusheight, µm	177.826 ± 11.860	292.911 ± 7.421 **
	Crypt depth, µm	166.355 ± 6.374	179.987 ± 10.119
	VCR	1.067 ± 0.038	1.680 ± 0.076 **

Note: data are presented as mean ± SEM deviation (n = 3), * 0.01 < *p* < 0.05, ** *p* < 0.01, *** *p* < 0.001. PBS represents the control group.

## Data Availability

The data generated and analyzed during this study are available from the corresponding authors on reasonable request.

## Abbreviations

The following abbreviations are used in this manuscript:

OTA	Ochratoxin A
OTB	Ochratoxin B
OTC	Ochratoxin C
GS	β-1,3-glucan synthase
COG	Clusters of Orthologous Genes
GO	Gene Ontology
KEGG	Kyoto Encyclopedia of Genes and Genomes
KAAS	The KEGG Automatic Annotation Server
CAZymes	The carbohydrate-active enzymes
HE	hematoxylin and eosin
OTUs	operational taxonomic units
BGCs	biosynthetic gene clusters
NRPS	non-ribosomal peptide synthetases
PKS	polyketide synthase
MDA	malondialdehyde
GSH-PX	glutathione enzyme
SOD	superoxide dismutase
VCR	villus-length-to-crypt-depth ratio
